# Structurally Oriented Nano-Sheets in Co Thin Films: Changing Their Anisotropic Physical Properties by Thermally-Induced Relaxation

**DOI:** 10.3390/ma10121390

**Published:** 2017-12-05

**Authors:** José Vergara, Cristina Favieres, César Magén, José María de Teresa, Manuel Ricardo Ibarra, Vicente Madurga

**Affiliations:** 1 Laboratorio de Magnetismo, Departamento de Física, Universidad Pública de Navarra, 31006 Pamplona, Spain; favieresc@unavarra.es (C.F.); vmadurga@unavarra.es (V.M.); 2 Instituto de Materiales Avanzados, Universidad Pública de Navarra, 31006 Pamplona, Spain; 3 Instituto de Nanociencia de Aragón (INA) and Laboratorio de Microscopías Avanzadas (LMA), Universidad de Zaragoza, 50018 Zaragoza, Spain; cmagend@unizar.es (C.M.); deteresa@unizar.es (J.M.d.T.); ibarra@unizar.es (M.R.I.); 4 Departamento de Física de la Materia Condensada, Universidad de Zaragoza, 50009 Zaragoza, Spain; 5 Instituto de Ciencia de Materiales de Aragón (ICMA), CSIC-Universidad de Zaragoza, 50009 Zaragoza, Spain

**Keywords:** nanocrystals, anisotropy resistivity, magnetic, optic, morphological, nano-sheets, STEM-EELS, structural relaxation, activation energy, laser deposition PLD

## Abstract

We show how nanocrystalline Co films formed by separated oblique nano-sheets display anisotropy in their resistivity, magnetization process, surface nano-morphology and optical transmission. After performing a heat treatment at 270 °C, these anisotropies decrease. This loss has been monitored measuring the resistivity as a function of temperature. The resistivity measured parallel to the direction of the nano-sheets has been constant up to 270 °C, but it decreases when measured perpendicular to the nano-sheets. This suggests the existence of a structural relaxation, which produces the change of the Co nano-sheets during annealing. The changes in the nano-morphology and the local chemical composition of the films at the nanoscale after heating above 270 °C have been analysed by scanning transmission electron microscopy (STEM). Thus, an approach and coalescence of the nano-sheets have been directly visualized. The spectrum of activation energies of this structural relaxation has indicated that the coalescence of the nano-sheets has taken place between 1.2 and 1.7 eV. In addition, an increase in the size of the nano-crystals has occurred in the samples annealed at 400 °C. This study may be relevant for the application in devices working, for example, in the GHz range and to achieve the retention of the anisotropy of these films at higher temperatures.

## 1. Introduction

The deposition of films at oblique angles of incidence has become an object of interest since novel applications were observed in them because of their particular nano-morphology based on nano-columns for some cases [1]. Thus, because of their high porosity, obliquely deposited indium tin oxide (ITO) was used in photoelectronic applications [2], as an antireflective coating in photonic cells [3] and for the detection of NO_2_ [4]. In addition, nano-columnar films may be used in devices working in the GHz range [5,6], as a consequence of the presence of a magnetic anisotropy in the plane of the films. Testing the temperature range where the magnetic anisotropy is present in the obliquely deposited films is relevant for their application in the GHz range.

The particular geometry of the nano-columnar films was tailored to different shapes such as vertical [7], zigzag [8] or nano-stripes [9], depending on the deposition characteristics. The presence of these nano-columns generated the anisotropic behaviour of the magnetic [10,11,12,13], optical [14,15], and transport properties [16,17,18]. For all these cases, no uniaxial magnetic anisotropy in the film plane was shown.

We developed particular oblique deposition geometry to grow thin films consisting of oblique nano-sheets directed towards the direction of the incoming flux of particles and perpendicular to the plane of incidence of the plasma. These nano-sheets were separated from the neighbouring ones by regions of free space, according to transmission electron microscopy (TEM) analysis [19]. A clear and well-defined uniaxial magnetic anisotropy was present in these films arising from this particular nano-morphology based on oriented and parallel nano-sheets [19]. However, on increasing the temperature, this nano-morphology relaxed, giving rise to isotropic physical properties [19]. In this work, we have measured the effect of the temperature on the physical properties of the obliquely deposited Co films with nano-sheets. We have measured the resistivity of the samples as a function of the temperature and have also studied various physical properties of as-deposited and annealed Co samples, through X-ray diffraction patterns, micrographs from TEM, surface morphology, magnetic hysteresis loops and transmittance of light.

## 2. Experimental Methods

Obliquely deposited (θ = 55°) nano-sheets-formed Co films have been obtained by pulsed laser deposition (PLD). A more detailed description of the experimental conditions was given in [19]. We have used a Nd-YAG (Quantum Brilliant) laser whose wavelength is 1064 nm. The frequency of the pulses has been 20 Hz and their duration is 4 ns. The energy at the target has been 220 mJ/pulse and the area of the laser impact ≈12 mm^2^. The laser beam has been at an angle of 45° with respect to the target and the emerging plasma has had the direction perpendicular to the target. The target consisted of a pure Co disk (99.99% purity) 20 mm in diameter that has rotated during deposition at 32 rpm. The films have been deposited both on Si (111) single crystals and on glass substrates. In the oblique deposition geometry, the substrates were attached to the lateral surface of a cone and the normal to the substrates has formed θ = 55° with respect to the plasma direction. The rotation symmetry axis of the cone was parallel to the plasma direction. This cone can be static or rotating with respect to this axis during deposition to increase the homogeneity of the film [19,20,21,22,23,24]. In addition to the obliquely deposited films, we have also deposited Co films in normal deposition geometry θ = 0° in order to highlight the difference in the physical properties between the Co films deposited in the oblique or normal geometry. The vacuum during the deposition was on the order or 10^−5^–10^−6^ mbar. The thickness of the samples has been typically 90 nm, as determined by a quartz crystal microbalance.

The surface nano-morphology of the samples has been imaged by means of a Metris 1000 (Burleigh, Fishers, NY, USA) scanning tunnelling microscope (STM) and a Nanotec atomic force microscope (AFM) [25]. The X-ray diffraction patterns have been measured with a 3000 TT X-ray diffractometer (Seifert, Ahrensburg, Germany). The magnetic moment of the samples has been measured with an EG&E (Gaithersburg, MD, USA) vibrating sample magnetometer (VSM). The optical anisotropy of the samples has been measured in a homemade optical ellipsometer. The resistance of the samples has been determined by the four-point probe method in a homemade device that was inserted inside a furnace. The temperature of the samples could thus be increased to 500 °C, at a controlled heating rate, while measuring the resistance simultaneously. The samples have been annealed at 270 °C and at 450 °C with a heating rate of 0.17 K/s. They have been kept at the maximum temperature for 1 min.

Local microstructure and chemical composition at the nanoscale have been analysed by STEM imaging in high angle annular dark field (HAADF) mode in combination with electron energy loss spectroscopy in a probe corrected FEI Titan microscope (Hillsboro, OR, USA) operated at 300 kV.

## 3. Results and Discussion 

**Figure 1:**
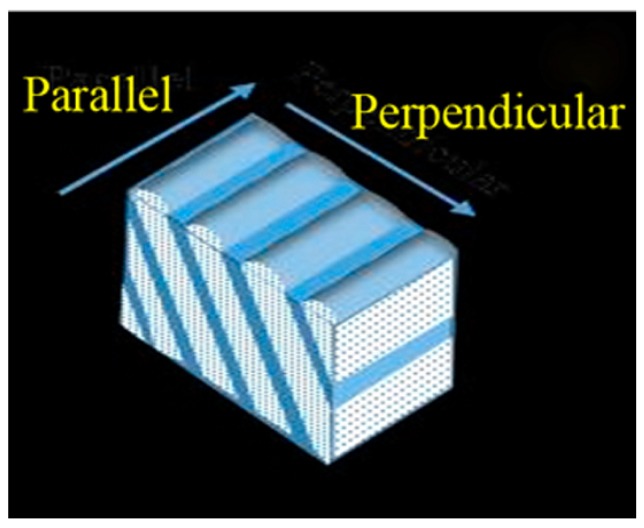
Drawing of the nano-morphology of the obliquely deposited Co film. The directions parallel and perpendicular, for the resistivity measurements, are shown.

From our samples (schematically represented in Figure 1 [19]), we have obtained the following results.

### 3.1. Resistivity

**Figure 2:**
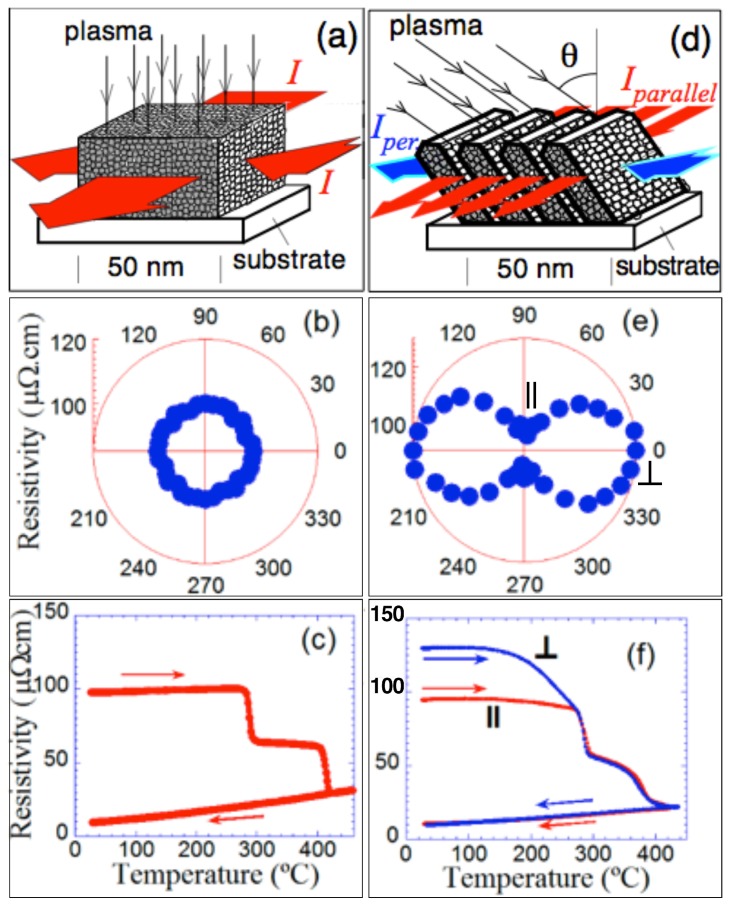
Scheme of the film with nano-crystals of 1 to 2 nm: (**a**) normally deposited; (**b**) polar plot of its resistivity at room temperature; (**c**) temperature dependence of its resistivity. Scheme also with nano-crystals of 1 to 2 nm: (**d**) corresponding to the Co film formed by nano-sheets; (**e**) polar plot of its resistivity at room temperature; and (**f**) temperature dependence of its resistivity. The zero angle value at the polar plots corresponds to the perpendicular to nano-sheets direction.

We have compared the transport behaviour of our obliquely deposited nano-sheets-formed Co films with the normally deposited ones, θ = 0°, Figure 2a,d. 

The normally deposited Co films are isotropic in the film plane. This fact is a consequence of their particular nano-morphology where Co nano-crystals (whose sizes range from 1 to 2 nm) are isotropically distributed in the film [19] (cf. Figure 2a). Consequently, when measuring resistivity in the film plane, an isotropic result has been obtained (see Figure 2b). On increasing the temperature, two steep drops in the isotropic resistivity of the normally deposited Co films has been measured at 270 °C and 410 °C, corresponding to irreversible processes (cf. Figure 2c).

On the contrary, the obliquely deposited nano-sheets-formed Co film has exhibited an anisotropic resistivity in the film plane as a consequence of these nano-sheets, Figure 2d, (although each nano-sheet consists of crystals of 1–2 nm as the normally deposited and isotropic film). Figure 2e shows a polar plot of the resistivity as a function of the measured in-plane angle. Previous works reported cases where the anisotropic conductivity was a consequence of an anisotropic morphology, e.g., nano-columns [18] and nano-wires [26,27], being cases different to our case. Our nano-sheets-formed Co films, in the as-deposited state, have shown a large resistivity (roughly 130 mOhm.cm), when the electric current has flowed along the perpendicular direction of the surface nano-strings (cf. Figure 1 and Figure 2f). However, on measuring the resistivity parallel to the nano-sheets, the resistivity of the as-deposited sample has been approximately 30% smaller. This large difference has reinforced the fact of the critical influence of the morphology due to nano-sheets on our samples’ behaviour versus the influence of the nano-columnar morphology corresponding to other works. Thus, in the temperature range from 30 to 270 °C, the resistivity measured parallel to the nano-sheets has been roughly constant. In contrast, the resistivity measured in the perpendicular direction has been initially constant up to 160 °C, but it has decreased continuously from its initial value of 130 mOhm.cm to its value at 270 °C, 90 mOhm.cm. From that temperature, the values of the resistivity measured both parallel and perpendicular became approximately equal, indicating that the resistivity after annealing on 270 °C of the Co sample formed by nano-sheets was then isotropic (cf. Figure 2f).

Just at 270 °C, both films’ resistivity has a sharp decrease (similar to that shown by the resistivity of the perpendicularly deposited film), indicating an irreversible process produced by a relaxation of the nano-crystalline structure: the normally deposited films lack nano-sheets, but nano-crystals also form their structure. Furthermore, on increasing the temperature to approximately 360 °C, another sharp decrease in resistivity has been measured. This temperature is lower than the corresponding temperature for the perpendicular deposited film (410 °C). On decreasing the temperature back to room temperature, the resistance also decreases but not in a reversible way, which was indicative of the fact that a structural relaxation or an increase of the nano-crystals’ size have taken place in the sample.

These changes in the resistivity of the obliquely deposited Co samples have been correlated with further results corresponding to the structure and nano-morphology of these samples, as we show below.

### 3.2. X-ray Diffraction

**Figure 3:**
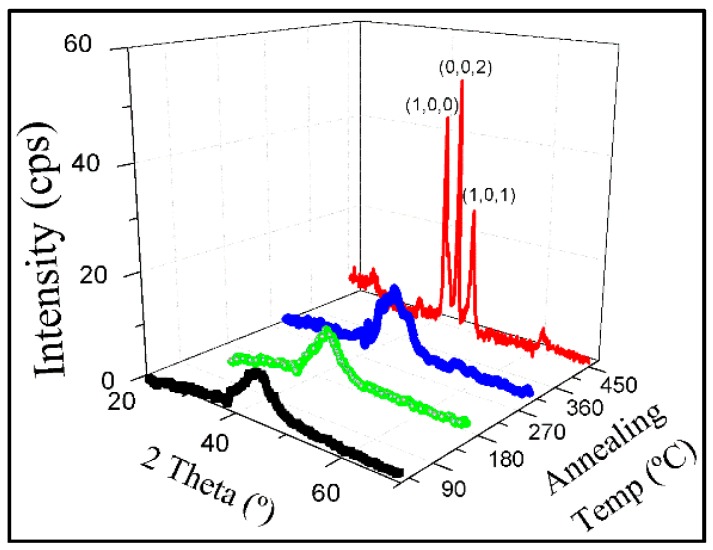
X-ray diffraction patterns of the obliquely as-deposited Co film (black), as well as annealed at 180 °C (green), 315 °C (blue) and 450 °C (red, in the coloured version). The indexes correspond to hcp Co.

The X-ray diffractogram of the as deposited Co sample at 55° do not show any crystalline peaks but a wide halo for a 2 theta value near 40 degrees, which has suggested the existence of an amorphous or nano-crystalline structure, cf. Figure 3.

The shape of the diffraction pattern does not change upon performing heat treatments up to 315 °C. Thus, in spite of the fact that the electrical resistance has decreased, a significant change in the crystal structure of the samples does not seem to occur, since the sample has retained an amorphous or nano-crystalline structure. In any case, relaxation processes have occurred in the film that has been deduced from the irreversible changes that have been observed in the values of its resistivity. After a further increase of the annealing temperature to 450 °C, the X-ray diffraction pattern of the annealed sample has shown the characteristic maxima that has corresponded to the hexagonal phase. In Figure 3, we have plotted all X-ray diffraction patterns of the nano-sheets-formed Co sample annealed at progressively higher temperatures. We have also performed several X-ray diffraction measurements with the incoming flux of X-ray photons either parallel, perpendicular or at 45°, with respect to the direction of the nano-sheets in our samples. All the results have been similar, which has indicated that no preferred crystalline orientation or texture has been detected in these samples from the viewpoint of the information extracted from X-ray diffractometry.

### 3.3. STM Studies: Surface Nano-Morphology

**Figure 4:**
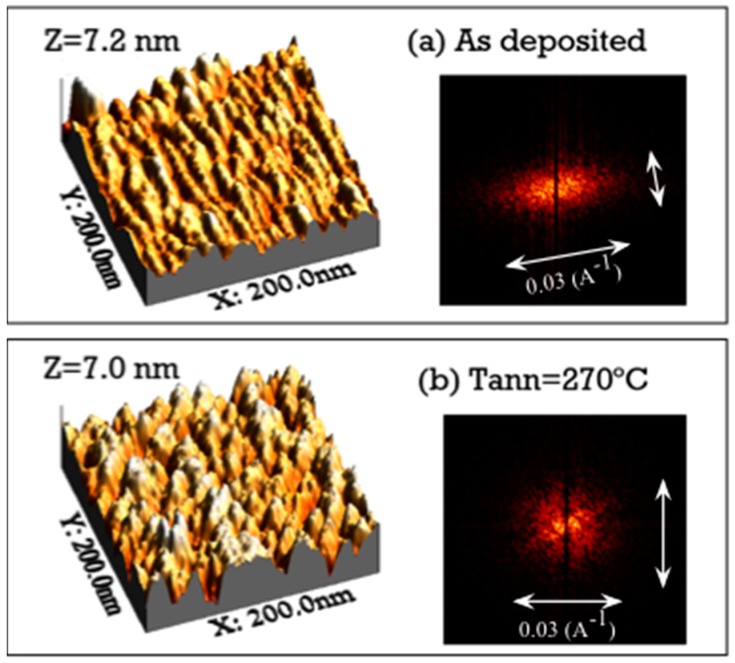

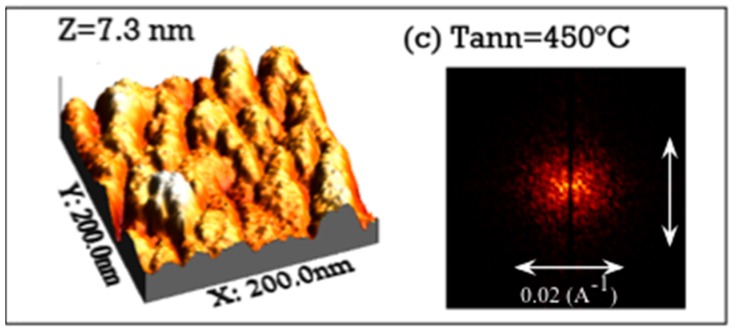
STM micrographs and 2D-FFT images of the obliquely deposited Co samples: (**a**) as-deposited; (**b**) 270 °C annealed and (**c**) 450 °C annealed.

The surface nano-morphology of the obliquely deposited Co samples has been measured by scanning tunnelling microscopy (STM), both in the as-deposited state and after various annealing processes. In the as-deposited state, Figure 4a, the Co sample has shown the presence on its surface of elongated nano-strings perpendicular to the plane of incidence of the plasma [22]. The average width of the nano-strings measured by STM (≈10 nm) has been similar to the medium value of the width of the crest of the nano-sheets at the top surface of the film (≈9.5 nm) according to TEM image that will be shown later in this work. The 2D fast Fourier transform (FFT) [25] of the STM image of the as-deposited film shows an elongated feature perpendicular to the surface nano-strings.

On annealing the samples at 270 °C, which is the temperature where the anisotropy in the resistivity vanished, the anisotropic pattern has disappeared from the STM image as well, cf. Figure 4b. An isotropic distribution of spots has been observed on the surface of the Co film, which was also confirmed by the circular image of the 2D-FFT of the STM micrograph, in contrast with the elongated image corresponding to the as-deposited sample. 

After annealing the sample at 450 °C, we still have observed on the surface of the samples an isotropic distribution of spots, cf. Figure 4c. Furthermore, the size of these spots has increased. This could be a consequence of the increase in the nano-crystals’ size that started at ≈410 °C. The 2-D FFT of the image has been roughly circular. An analysis of the surface of the previous three images reveals that the roughness does not increase as a consequence of the annealing process, but it remains constant at approximately 1 nm.

### 3.4. Optical Anisotropy

**Figure 5:**
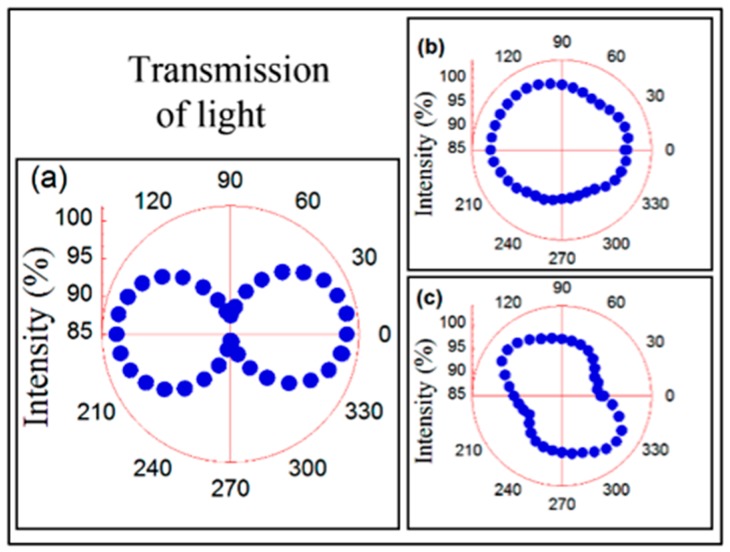
Polar plots of the transmitted intensity of polarized light for the obliquely deposited Co samples: (**a**) as-deposited; (**b**) 270 °C annealed and (**c**) 450 °C annealed. The 0 angle value corresponds to the direction of polarization parallel to the nano-sheets direction.

The transmission of polarized red light (λ = 650 nm) through an obliquely deposited Co film has depended also on the nano-morphology of the film. For the normally deposited Co samples, the transmission of polarized light through it was isotropic [28]. In contrast, for the nano-sheets-formed Co sample, the transmission of polarized light through the sample has been anisotropic, cf. Figure 5a. This fact has been a consequence of its anisotropic nano-morphology: the intensity of the light transmitted through the sample has depended on the angle between the direction of the plane of polarization of the light and the direction of the nano-sheets in the Co film.

However, upon annealing the sample at 270 °C, the transmission of light through the sample became isotropic, cf. Figure 5b. Furthermore, increasing the annealing temperature to 450 °C, again, the transmission of light through the crystalline Co film has been roughly isotropic, cf. Figure 5c.

### 3.5. Magnetic Anisotropy

The particular nano-morphology of our obliquely deposited Co films, the existence of obliquely oriented nano-sheets, is the origin of its uniaxial magnetic anisotropy [19]. The direction of the nano-strings on their surface is the easy direction of magnetization in the film. Hysteresis loops corresponding to the field applied in the film plane and along this direction correspond roughly to a bi-stable behaviour. Upon applying a magnetic field in the film plane and perpendicular to the previous direction, a pure rotation process of the magnetization occurs.

**Figure 6:**
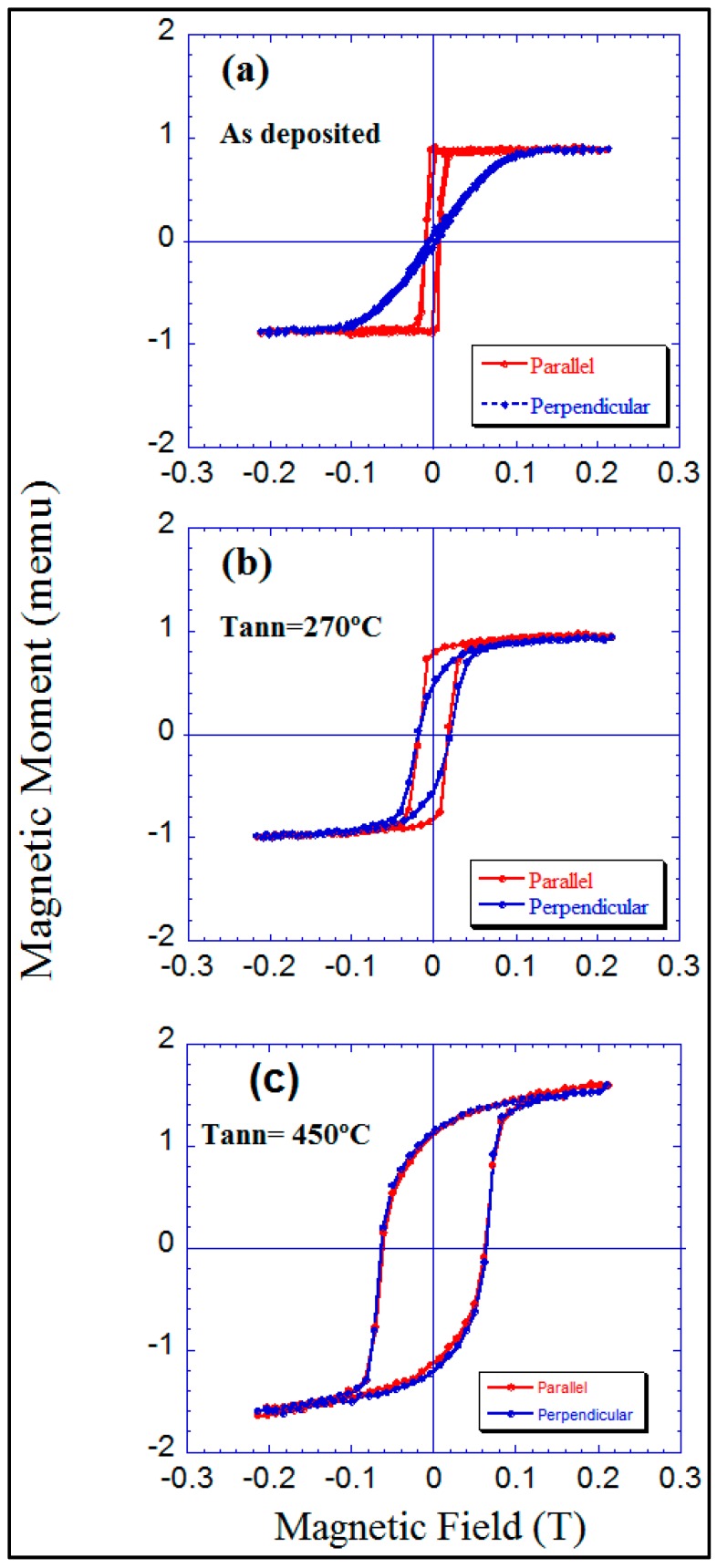
Hysteresis loops measured at room temperature parallel and perpendicular to the direction of the nano-sheets of the Co samples: (**a**) as-deposited sample; (**b**) 270 °C annealed; (**c**) 450 °C annealed.

Therefore, an in-plane uniaxial magnetic anisotropy is present in these samples. Both hysteresis loops along the former directions and for the as-deposited sample are displayed in Figure 6a. However, upon annealing the film at 270 °C, the uniaxial magnetic anisotropy has been lost, cf. Figure 6b. By annealing at 450 °C, the Co sample shows no magnetic anisotropy, cf. Figure 6c, but an increase in its magnetic moment. This increase might be related to the long-range order modification appearing in the annealed sample because of the growth of the nano-crystals.

### 3.6. TEM Studies

**Figure 7:**
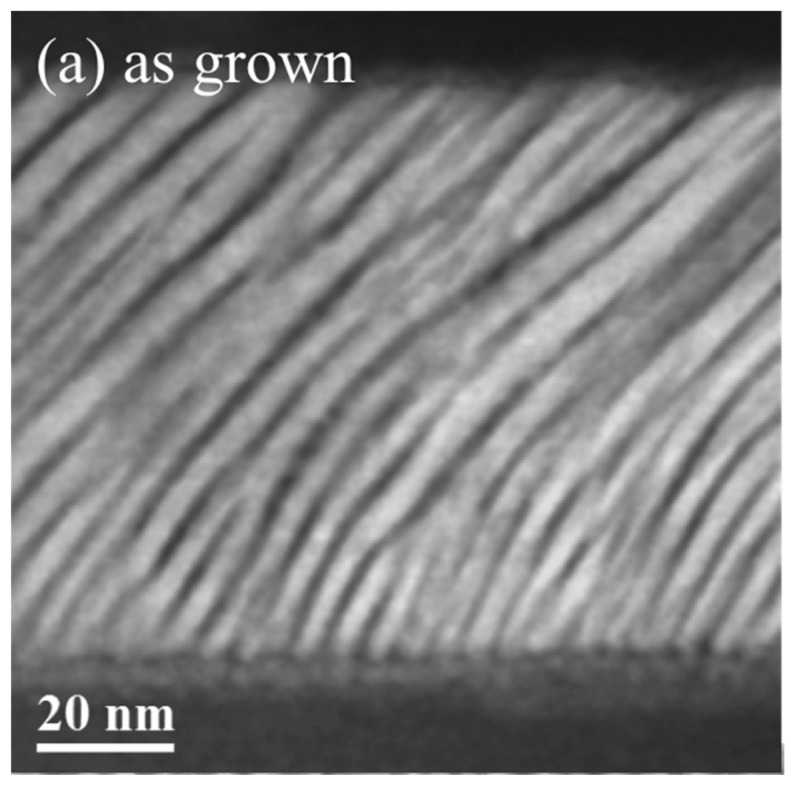

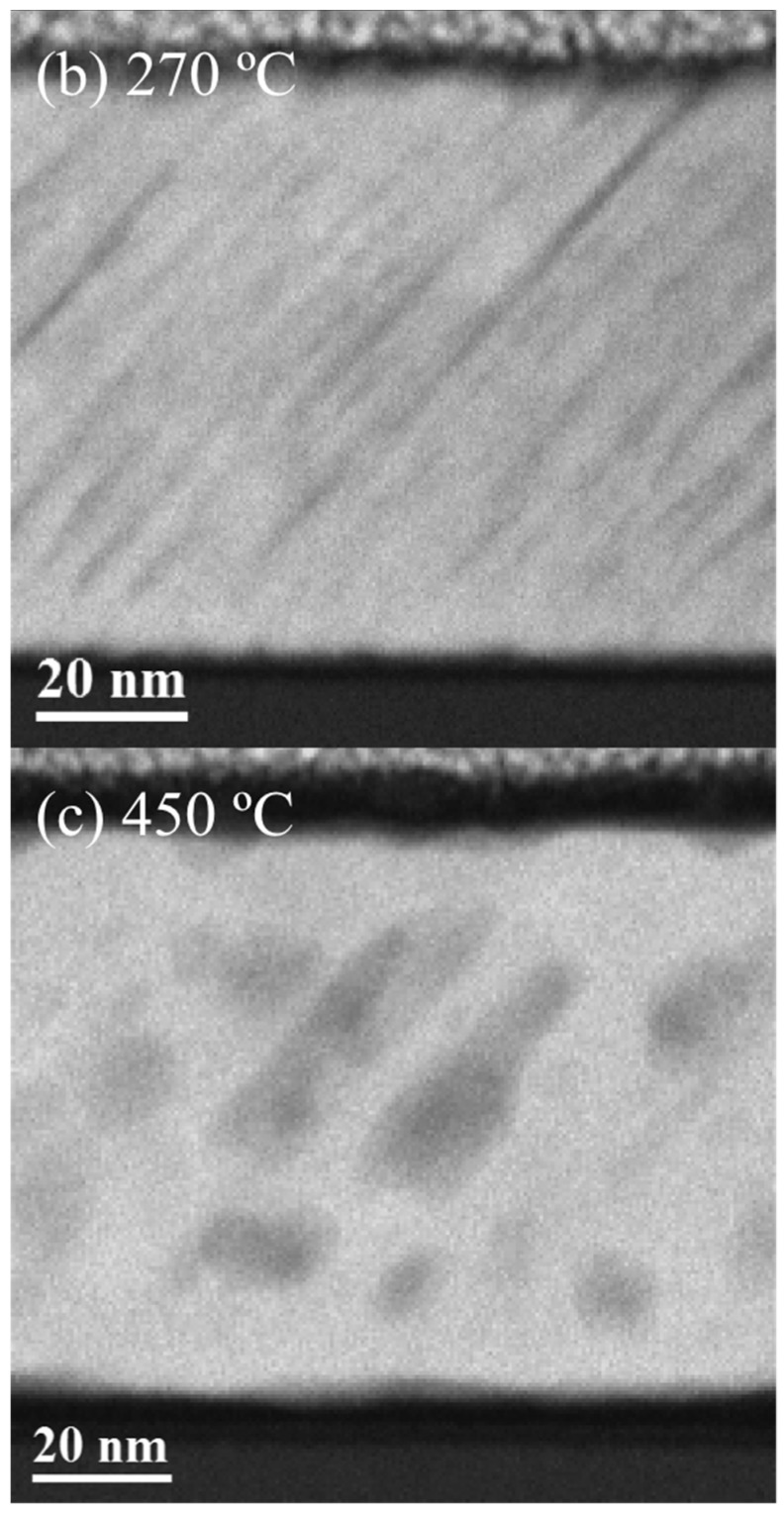
Cross-sectional HAADF-STEM images of an obliquely deposited Co film: (**a**) in the as-deposited state; (**b**) annealed at 270 °C and (**c**) annealed at 450 °C.

STEM images collected on the as-deposited samples display a regular pattern of oblique Co nano-sheets, as we had shown before in [19], whose thickness ranged from approximately 4.5 nm in the regions close to the substrate to 7 nm near the surface of the deposit (cf. Figure 7a). These oblique Co nano-sheets are separated approximately 1–2 nm. After annealing the film at 270 °C (see Figure 7b), the film still displays a pattern of oblique Co nano-entities, but the separation between them decreases, and the nano-sheets tend to merge. Thus, at this annealing temperature of 270 °C, a coalescence-like process seems to occur: the nano-sheets of Co approach each other, reaching the contact between them in many cases. This nano-morphological process of the extinction of oriented nano-sheets, practically completed at 270 °C is simultaneous to the disappearance of the uniaxial anisotropic behaviour of various physical parameters, as we have shown in this work. Even though some physical properties change drastically, the films remain amorphous or nano-crystalline. The results of further annealing at 450 °C are illustrated in Figure 7c. No trace of the nano-sheets of Co can be found any more. According to the X-ray diffraction results corresponding to the 450 °C annealed sample, this TEM observation corresponds to the formation of an almost homogeneous hcp Co film.

### 3.7. Compositional Analysis by STEM-EELS

**Figure 8:**
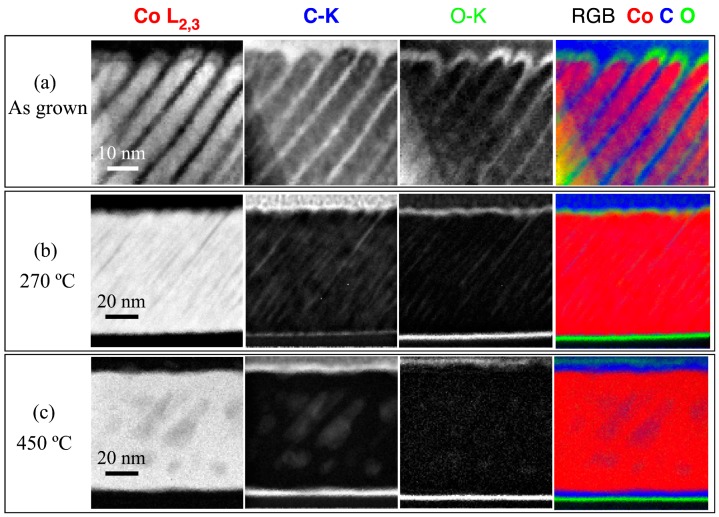
STEM-EELS chemical maps of an obliquely deposited Co film in the as-deposited state (**a**), annealed at 270 °C (**b**) and annealed at 450 °C (**c**).

The distribution of chemical elements within the nano-sheets has been analysed by STEM-EELS (Electron energy loss spectroscopy). Spectrum images of the as-grown samples, as well as the films annealed at 270 °C and 450 °C, are illustrated in Figure 8. The as-deposited Co film deposited at 55°, shown in Figure 8a, makes evident that the presence of Co nano-sheets formed the film. The regions between the nano-sheets, where no Co signal can be found, show the presence of C and, to a lesser extent, O. Both can be ascribed to the presence of the resin used for the preparation of the cross-sectional specimens. There is also an abundance of O on top of the nano-sheets, which is assumed to be the natural oxidation process that occurred after the extraction of the samples from the deposition chamber. The compositional mapping of the Co film annealed at 270 °C is shown in Figure 8b. The Co regions appear closer to each other. Some traces of C may still be observed between the oblique Co nano-sheets. Additionally, O is observed on the surface of the film and at the interface with the substrate, as a consequence of the oxidation of the Si wafer surface. No traces of other elements are found within the nano-sheets. Finally, Figure 8c displays the local compositional analysis of the Co sample annealed at 450 °C. In this sample, Co is again the main component of the film. No trace of the nano-sheet structure remains, except for small dispersed regions, where traces of C are embedded in the film.

### 3.8. Activation Energy

Nanostructured materials obtained through a rapid solidification process, such as pulsed laser deposition, are metastable with respect to structural relaxation or crystallization. In our films, the kinetic processes corresponded, either to atomic rearrangements (for instance the coalescence of the Co nano-sheets upon heating the samples) or to crystallization, when the annealing temperature was above 400 °C. The rate at which these kinetic processes take place can be equated in terms of the Arrhenius expression, i.e., the probability of these processes depends both on the temperature and on the activation energy.

Due to the particular nanostructure and nano-morphology of our obliquely deposited Co films, the energies involved in the structural relaxation lie within a wide range of energies, which is the spectrum of activation energies. This energy spectrum can be determined, according to the Primak’s model [29] either by isothermal annealing [30] or by annealing the samples at a constant heating rate. Within the framework of this model, the different processes in this spectrum of activation energies are achieved either when the temperature of the samples is increased or through temporal evolution (isothermal annealing).

In our samples, the activation energy spectrum of these rearrangements [29,30], *p*_0_(*E*), has been obtained, through annealing, from the derivative of the normalized resistivity (*ρ*(*T*)/*ρ*_0_) with respect to the temperature, according to References [29,31,32]:

(1) $$p_{0}\left(E_{0}\right)=-\frac{d\left(\frac{\rho \left(T\right)}{\rho_{0}}\right)}{dT}\frac{dT}{dE_{0}},$$

where *ρ*_0_ is the room temperature resistivity of the as obtained films, and *E*_0_ is the particular value of the energy that is activated at temperature *T*. The activation energy *E*_0_ and the temperature *T* accomplish the relationship [29]:

(2) $$\frac{E_{0}}{k_{B}T}+ln\left(\frac{E_{0}}{k_{B}T}+2\right)=ln\left(\frac{\upsilon_{o}T}{V_{S}}\right).$$

In the previous logarithmic equation, *ν*_0_ is the attack frequency that, in this work, we have chosen to be 10^12^ s^−1^ [31], close to the Debye frequency for single atom jumps, since the kinetic processes in these samples correspond to atomic displacements to free neighbouring positions. *V_s_* is the heating rate (0.17 K/s in our experiments). In order to obtain directly a value of the activation energy *E*_0_ as a function of the temperature *T*, logarithmic Equation (2) was solved, within the Primak’s model, through an approximation to a linear behaviour [29,31]:

*y* + *ln*(*y* + 2) ≈ *a* + *yb*, (3)

where the variable *y = E*_0_*/k_B_T*. For this fit, we have assumed a range of activation energies from 0.5 to 2.5 eV, corresponding to values of the activation energies found in amorphous materials [30,31]; meanwhile, the range of temperatures (300–1000 K) corresponded to the range of annealing temperatures of our experiment. The parameters *a* and *b* obtained from this approximation to a linear behavior are 2.6 and 1.03.

Consequently, the Equation (2) is transformed into:

(4) $$E_{0}=\frac{k_{B}T}{1.03}\left[ln\left(\frac{\upsilon_{o}T}{V_{S}}\right)-2.6\right],$$

which has allowed us to represent the spectrum of activation energies as a function of the derivative of the normalized resistivity with respect to temperature versus the activation energies.

**Figure 9:**
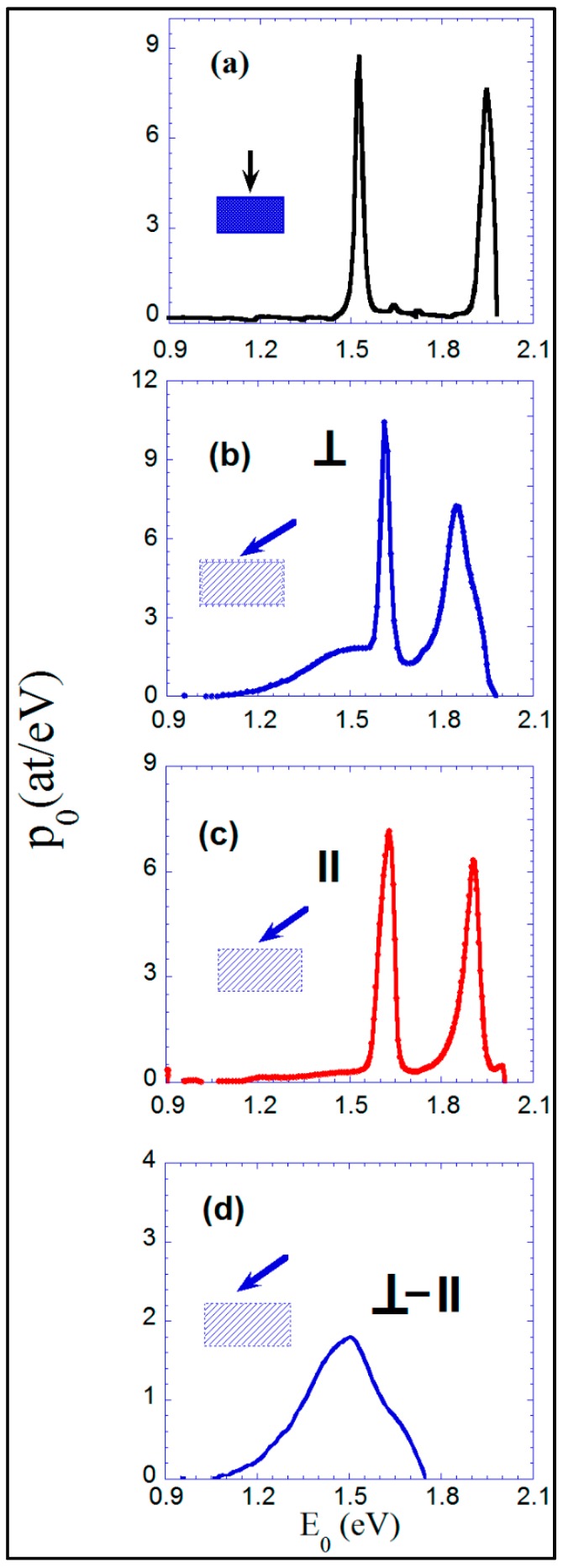
Activation energy spectrum obtained from the measurements of the resistivity: (**a**) of the normally deposited film; (**b**) of the nano-sheets-formed Co film measured along the perpendicular direction; (**c**) measured along the parallel direction; and (**d**) the difference between the two spectra corresponding to (**b**,**c**) representations.

We have determined the spectrum of activation energies first for a normally deposited isotropic Co film using the resistivity measurements (Figure 2c). This spectrum is shown in Figure 9a, and it has served as a reference.

In addition, we have obtained the activation energy spectrum for the nano-sheets-formed Co film deposited at 55°. We have made use of the temperature dependence of the resistivity, Figure 2f, measured both perpendicular to the direction of the nano-strings of the surface (cf. Figure 9b) and parallel to the nano-strings (cf. Figure 9c).

Both spectra have displayed similar characteristic peaks at activation energies corresponding to relaxation processes at 1.6 eV and at 1.9 eV, associated with irreversible changes in the resistivity at 283 °C and 375 °C, respectively. The energies involved in these processes have resulted in a similar energy range to previously reported relaxation processes in amorphous metallic alloys [31,32].

The spectrum corresponding to the resistivity measured perpendicular to the nano-strings, Figure 9b, has shown a broad maximum ranging from 1.2 to 1.7 eV (160 to 270 °C) (see Figure 9d), which has not been observed in the activation energy spectrum from the obliquely deposited Co sample measured parallel to the direction of the nano-strings (Figure 9c). This maximum has not been observed in the perpendicularly deposited sample (Figure 9a).

Comparing the spectrum of the perpendicular deposited film, Figure 9a, with the corresponding one of the obliquely deposited film and measured in the parallel direction, Figure 9c, the similarity can be observed between their shape and between the corresponding values for the two characteristic peaks of the irreversible processes. This can be explained by considering the similitude of the nanostructures of these two films (nano-crystals of 1–2 nm size), although one is isotropic in its plane and the other has anisotropically oriented nano-sheets. It can be deduced that the internal structure of these nano-sheets must be similar to the internal structure of the perpendicular deposited film, as the X-ray diffraction results showed [19].

The difference between the spectra corresponding to the perpendicular and parallel resistivities, from the same obliquely deposited sample, is displayed in Figure 9d. This difference can be explained as a consequence of the evolution of the nano-sheets when heating the film: the approaching of the nano-sheets observed by STEM. The resistivity measured parallel to the nano-sheets has not been affected by this approach: the current flows inside each nano-sheet, isolated or in contact. On the contrary, the resistivity measured perpendicular to the nano-sheets has been strongly modified by this approaching: from state (1): for the as-deposited sample with separated nano-sheets and “no conduction” between them except some nano-sheets in contact, Figure 7a, which participate in the conduction, until state (2): with practically all nano-sheets in-contact—therefore conductive between them—after heating to 270 °C. The usefulness of the anisotropic resistivity measurements of these films thus becomes evident.

## 4. Conclusions

The origin and mechanism of the loss of the physical anisotropy present in the oblique nano-sheets-formed Co films have been elucidated. The well-known magnetic anisotropy of these samples has been extended in this work to include the resistivity, surface nano-morphology and optical transmission anisotropies, as well as their simultaneous vanishing. After annealing the Co samples to 270 °C, the anisotropy in these physical properties has disappeared, even though no growth of the nano-crystals or crystallization processes of the as-deposited film have occurred at that temperature. This vanishing in anisotropy has been monitored through the measurements of the resistivity as a function of the temperature. Thus, while the resistivity parallel to the nano-sheets has remained nearly constant up to 270 °C, the resistivity perpendicular to the nano-sheets has decreased by roughly one third of its initial value. This fact has been simultaneous to a coalescence of the oblique Co nano-sheets, as has been evidenced by STEM. This merging process has improved the conduction mechanism in the direction perpendicular to the nano-sheets.

The loss of the anisotropy of the surface nano-morphology, the loss of the anisotropic transmission of light and the loss of the magnetic anisotropy have been justified with the same evidence of the approaching and contact of the nano-sheets (finally, they practically disappear, forming a continuous and homogeneous nano-crystalline film).

The spectrum of the activation energy of the processes participating in these approaching and coalescence phenomena of the nano-sheets has been established. It has ranged between 1.2 and 1.7 eV, exhibiting a maximum at approximately 1.5 eV.

Further heat treatments beyond 270 °C have resulted in two irreversible processes with two sharp decreases in the resistivity. The first one, whose activation energy peak has been found at 1.6 eV, has corresponded to some relaxation process in the nano-crystals or in the frontier between them, since no crystalline peaks have been found in the X-ray analysis. The second one, at approximately 1.9 eV, has corresponded to the growth of the nano-crystals that has been identified by means the X-ray diffraction peaks. In this case, there has been a more homogeneous distribution of the Co within the film as it has been revealed by the STEM-EELS image.

In addition, we have estimated the temperature range where the anisotropy continues to be present and, consequently, the range where the material might be used—for example, in devices working in the GHz range.
